# Tracking Mobile Sinks via Analysis of Movement Angle Changes in WSNs

**DOI:** 10.3390/s16040449

**Published:** 2016-03-29

**Authors:** Guisong Yang, Huifen Xu, Xingyu He, Gang Wang, Naixue Xiong, Chunxue Wu

**Affiliations:** 1 Department of Computer Science and Engineering, School of Optical-Electrical and Computer Engineering, University of Shanghai for Science and Technology, Shanghai 200093, China; gsyang@usst.edu.cn (G.Y.); huifenxu@st.usst.edu.cn (H.X.); gwang.buaa@gmail.com (G.W.); wcx@usst.edu.cn (C.W.); 2 Shanghai Key Lab of Modern Optical Systems, Shanghai 20093, China; 3 Public Experiment Center, University of Shanghai for Science and Technology, Shanghai 200093, China; 4 College of Electronic and Information Engineering, Tongji University, Shanghai 201804, China

**Keywords:** wireless sensor networks, tracking, mobile sinks, trajectory, movement angles

## Abstract

Existing methods for tracking mobile sinks in Wireless Sensor Networks (WSNs) often incur considerable energy consumption and overhead. To address this issue, we propose a Detour-Aware Mobile Sink Tracking (DAMST) method via analysis of movement angle changes of mobile sinks, for collecting data in a low-overhead and energy efficient way. In the proposed method, while a mobile sink passes through a region, it appoints a specific node as a region agent to collect information of the whole region, and records nodes near or on its trajectory as footprints. If it needs information from the region agent in a future time it will construct an energy efficient path from the region agent to itself by calculating its own movement angles according to the footprints, as well as getting rid of detours by analyzing these movement angles. Finally, the performance of the tracking method is evaluated systematically under different trajectory patterns and footprint appointment intervals. The simulation results consolidate that DAMST has advantages in reducing energy consumption and data overhead.

## 1. Introduction

Wireless Sensor Networks (WSNs) consist of a large number of distributed sensor nodes, which are widely applied for data collection. To make WSNs practical, many problems need to be properly handled, including limited network life due to limited energy storage, low connectivity under a low node density, and especially the hotspot problem causing early death of nodes and network partition. To address these problems, WSNs with mobile sinks have been explored for their potential positive effects on prolonging network lifetime and even collecting data in a sparse network.

However, in WSNs with mobile sinks, the traditional routing protocols for static sinks are no longer applicable and new challenges emerge because the location information of mobile sinks changes dynamically. It has been shown that the mobility of sinks can provide advantages for data collection, therefore, some studies concentrate more on how to control the trajectories of mobile sinks [1,2,3]. The limitation of these studies is that mobile sinks are assumed to move along predetermined routes in most of the cases, which is not practical in real WSNs. To achieve a more flexible application, some data collection protocols have been proposed to allow mobile sinks to move randomly [4,5], which however requires a large amount of energy and data overhead for announcing the location information of the mobile sinks. SinkTrail [6] is a typical mobile sink tracking method allowing random mobility of mobile sinks. In SinkTrail, trail messages need to be broadcast to each network node and the trail reference information of each node needs to be updated after each movement of the mobile sink. The frequent broadcasting of trail message and the updating of trail references results in a large amount of overhead, network congestion and could reduce the network lifetime due to high energy consumption [7].

**Figure 1:**
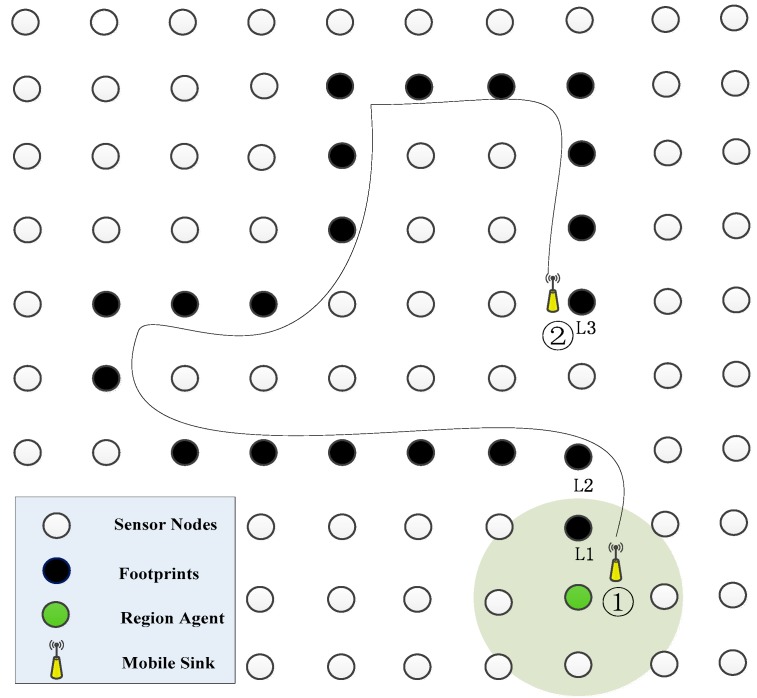
A typical scenario for DAMST.

In this paper, Detour-Aware Mobile Sink Tracking (DAMST), an energy efficient mobile sink tracking method with movement angle changes analysis, is developed for lowering energy consumption and data overhead. Figure 1 shows a typical scenario for DAMST. In Figure 1, while a mobile sink passes through a region (the circular shadow region in Figure 1), and in case that it considers the information of this region should be transferred at some future time (the scale of the region could be decided by the mobile sink), it will firstly appoint a node in this region as a Region Agent (RA). The RA is responsible for collecting the data of the whole region. Then, the mobile sink chooses nodes on or near its trajectory as footprints and records their location information. When the mobile sink needs information from the RA at some time, it will send a data query to the RA and get a reply from the RA along the movement trajectory marked by the footprints.

However, we notice that sometimes the moving trajectory of the mobile sink (the black curve in Figure 1) may take too many detours and not be suitable for data collection over a long time range. Therefore, to obtain an energy efficient data transition path from the RA to the mobile sink and reduce data overhead spent on storing the location information of useless footprints, DAMST is designed to help mobile sinks delete footprints on the detours of the moving trajectory by analyzing movement angle changes from one footprint to another.

In Figure 1, after the mobile sink moves from a RA (Node 1) to a current node (Node 2) along a trajectory (the black curve), by analyzing the movement angle changes on the trajectory, the mobile sink can effectively cancel most of the location information of the footprints on detours, only reserve location information of three useful footprints L1, L2, and L3, and further establish an energy efficient data transition path consisting of the rest footprints L1, L2, and L3 from the RA to the current node.

The main contributions of this work may be summarized as follows:

1. A new detour-aware mobile sink tracking method via analysis of movement angle changes is proposed, which is beneficial for establishing energy efficient data transition path from a RA to a mobile sink without detours, and decreasing overhead for normal nodes and the mobile sink.
2. A routing algorithm based on route deviation angles is designed to construct a shortest path between two unconnected adjacent useful footprints, which can promote the data transition speed and further reduce the overhead.

Moreover, in DAMST, it is not necessary for normal sensor nodes to be equipped with GPS devices to track mobile sinks, and a novel packet format is defined for the mobile sinks to conveniently define data type and data collection scope. The performance of DAMST on different trajectory patterns is simulated and explored. The simulation results verify the significant advantages of DAMST in obviously reducing the network energy consumption and overhead.

The rest of this paper is organized as follows: In Section 2, related works are introduced. Design details of DAMST are presented in Section 3. The performance of DAMST is analyzed and investigated in Section 4, and Section 5 concludes our work.

## 2. Related Work

Since mobile sinks are flexible to move around and facilitate data gathering efficiency, they have been widely used in WSNs for data collection. To better benefit from mobile sinks’ mobility, a lot of work has been done recently on the trajectories of mobile sinks in WSNs. These studies can be classified into three categories [8] based on the trajectory patterns of the mobile sinks: Controlled trajectory, random trajectory, and predicted trajectory.

The first category of protocols requires mobile sinks to move along controlled trajectories [9,10,11,12,13]. In [9], a mobile sink moves along fixed trajectories and divides a monitored region into two parts, the Direct Communication Area (DCA) between the trajectories and the Multi-hop Communication Area (MCA) for far-off sensors, and the nodes within the MCA are assigned to the corresponding subsinks within the DCA according to the length of the communication time between the mobile sink and the subsinks. In [10], the authors developed a basic smooth path construction algorithm based on the traveling salesman problem, which can reduce the physical collection delay while achieving other performance improvements. In [11], Ghafoor and Rehmani proposed a novel approach for mobile sink tracking based on a Hilbert Space Filling Curve which changes the curve order according to node density. These protocols can prolong network lifetime and reduce energy consumption, but have less flexibility due to the constrained trajectories of the mobile sinks. In our work, DAMST allows mobile sinks to move randomly.

TTDD [14] is one of the protocols in the second category which uses randomly moving sinks to gather data. In TTDD, a grid structure is built among dissemination nodes and mobile sinks which constantly broadcast their location information and data queries along the dissemination nodes. SinkTrail [6] is another protocol in the second category, in which a mobile sink also needs to broadcast a trail message to each node, and a unique logical coordination of each node is updated according to the new trail message. In these two protocols, the continuous propagation of mobile sink location information throughout the whole network leads to high energy consumption and increased overhead. In DAMST, the trail message of a mobile sink is just sent along a path that is optimized via analysis of movement angle changes instead of broadcast to the whole network, which consumes less energy and has lower data overhead.

Different from the aforementioned two categories of researches on mobile sinks, the third category focuses on predicting a sink’s trajectory [15,16]. In [15], Lee and Wicke developed an approach for sending data to relay nodes along a predicted trajectory. In [16], routing states in the network are pre-computed and stored using a mobility graph. The predicted paths are however not accurate and may result in low data delivery rates.

In our research, being aware of the fact that the movement angles could play important roles in tracking systems [17,18], DAMST is designed to detect detours on the trajectory of a mobile sink by comparing movement angles between two sequential footprints, and reserve useful footprints by getting rid of those footprints on the detours. Furthermore, while constructing an appropriate data transition path among the useful footprints, it may happen that the distance between two adjacent useful footprints is greater than the node communication radius, in this case, DAMST tries to select appropriate relay nodes between the two unconnected adjacent footprints.

## 3. Details of DAMST

### 3.1. Network Model

A network with lots of sensor nodes and several mobile sinks scattered uniformly in an area is considered. The sensor nodes communicate with each other via radio links. The whole network is assumed to be fully connected. The mobiles sinks have unlimited energy, large storage space and powerful processing capability to gather data from sensor nodes of interest. The mobile sinks are capable of obtaining real-time location information via their own GPS devices. However, the sensor nodes are not necessarily supported by the GPS function. While moving, the mobile sinks appoint some sensor nodes as RAs and some other sensor nodes on or near their trajectories as footprints. Each node in the network has a unique ID which is used as the routing address. For a clear description without loss of generality, the scenario where there is only one mobile sink and one RA is mainly discussed in the following elaboration.

### 3.2. DAMST Point List and Packet Format

#### 3.2.1. DAMST Point List and DAMST Angle List

As shown in Figure 1, if a mobile sink is interested in the status of a region, it appoints a node in the region as a RA while it passes by the RA. The RA is responsible for collecting data in the region of interest. After leaving the region, the mobile sink periodically chooses nodes on or near its trajectory as footprints, obtains their location information by its own GPS and records their ID and location information. If a time interval to designate two successive footprints is set as *t*, a communication radius of each node is set as *r*, and a speed of the mobile sink is set as *v*, these parameters are required to satisfy the following inequality:

t × v < r(1)

The mobile sink’s movement angles are called as DAMST angles and defined in Section 3.3.1. While the mobile sink moves, its DAMST angles are recorded in a DAMST angle list and its footprints are put into a DAMST point list as DAMST points. After each footprint appointment, the mobile sink puts the new footprint into the DAMST point list, and calculates a new DAMST angle then puts it into the DAMST angle list. Meanwhile, for obtaining an energy efficient path to the RA, the mobile sink cancels DAMST points on detours via analysis of the DAMST angles in the DAMST angle list. After that, both the DAMST point list and the DAMST angle list need to be updated.

#### 3.2.2. Packet Format

Once the mobile sink needs the sensing data of the interested region, it sends a data query to the RA and gets a reply via DAMST points on the trajectory from the RA. Both the query packets and the reply packets follow the packet format defined in Table 1. In the packet format, packet type represents that a packet is a query packet or a reply packet. The RA ID points out which RA the mobile sink requests to. Data type, collection period and node collection radius of the region are designated by the mobile sink to indicate what kind of data it needs, how often to update data and how large of the interested region. The mobile sink can require the RA to send data of a specific node through Node ID.

In a scenario with multiple mobile sinks, the Sink ID informs the RA which one needs the interested data. The most important two parameters in the packet format are DAMST point list and route list, which determine how to make routing decisions in forwarding the query packets and reply packets, the detailed parts are explained in Section 3.3.2. Before delving into other details of DAMST, we define some important notations in Table 2.

### 3.3. Updating DAMST Angle List and DAMST Point List

#### 3.3.1. Definition of DAMST Angles

**Figure 2:**
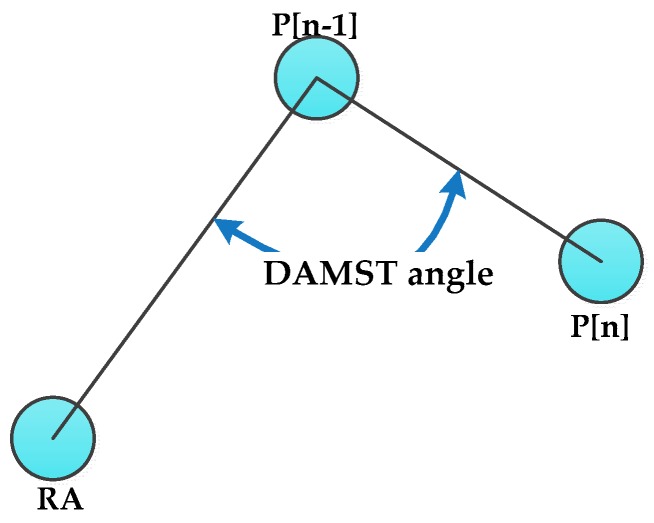
A DAMST angle.

As described in Section 3.2.1, once the mobile sink designates a new footprint, it immediately updates the DAMST point list and DAMST angle list. For detailed description of these two lists update, definition of DAMST Angles is firstly provided as follows in conjunction with Figure 2. There is a correspondence between the DAMST points and the DAMST angles, specifically, the (n)th DAMST point in the DAMST point list is a vertex of the (n)th DAMST angle in the DAMST angle list.

**Definition 1.**  The (n−1) th DAMST angle A[n−1] in the DAMST angle list is an angle between the edge from the (n−1)th DAMST point P[n−1] to a RA and the edge from the (n)th DAMST point P[n] to the (n−1)th DAMST point P[n−1], which is less than or equal to 180°.

After each footprint appointment, the new footprint becomes the last element in the DAMST point list and a new DAMST angle is calculated accordingly based on Definition 1 and then put into the DAMST angle list. How to cancel a DAMST point on a detour via analysis its corresponding DAMST angle is described in Section 3.3.2.

#### 3.3.2. Analysis of DAMST Angles

DAMST angles in the DAMST angle list reflect the trajectory changes of the mobile sink. The detours on the trajectory of the mobile sink can be found via analysis of them. The whole process includes many rounds of DAMST Angle analysis, and each round of it includes two phases: A single DAMST angle analysis phase and an adjacent DAMST angle analysis phase.

##### A. Single DAMST Angle Analysis Phase

**Figure 3:**
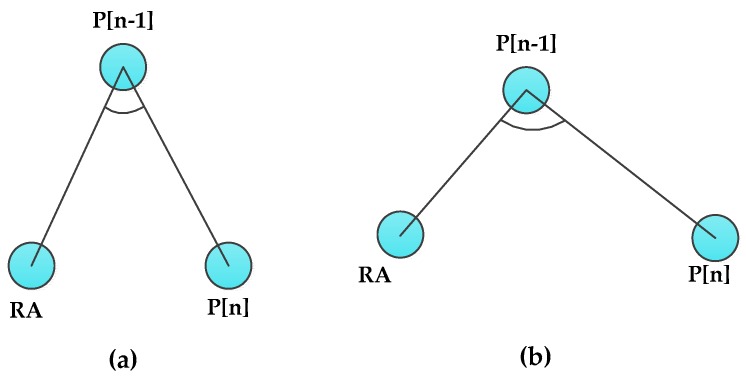
(**a**) Acute and (**b**) obtuse DAMST angles.

As shown in Figure 3, for a single DAMST angle, the acute angle shown in Figure 3a involves a longer detour than the obtuse one shown in Figure 3b. Therefore, in the single DAMST angle analysis phase, each DAMST angle is compared with 90°, the DAMST point whose corresponding DAMST angle is an acute angle should be cancelled from the DAMST point list. For example, if the (n−1)th DAMST angle in the DAMST angle list is less than 90°, the (n−1)th DAMST point needs to be cancelled.

**Figure 4:**
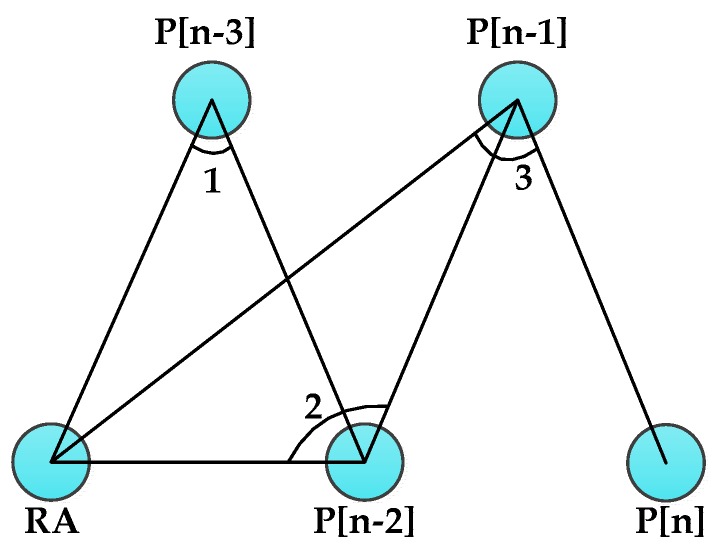
An example of single DAMST angle analysis.

For example, in Figure 4, because angles 1 and 3 are less than 90°, P[n−1] and P[n−3] are cancelled from the DAMST point list and P[n−2] and P[n] are reserved. At the end of this phase, some DAMST points on longer detours are cancelled from the DAMST point list, accordingly, the DAMST angles in the DAMST angle list needs to be recalculated according to the reserved DAMST points based on the Definition 1.

##### B. Adjacent DAMST Angle Analysis Phase

**Figure 5:**
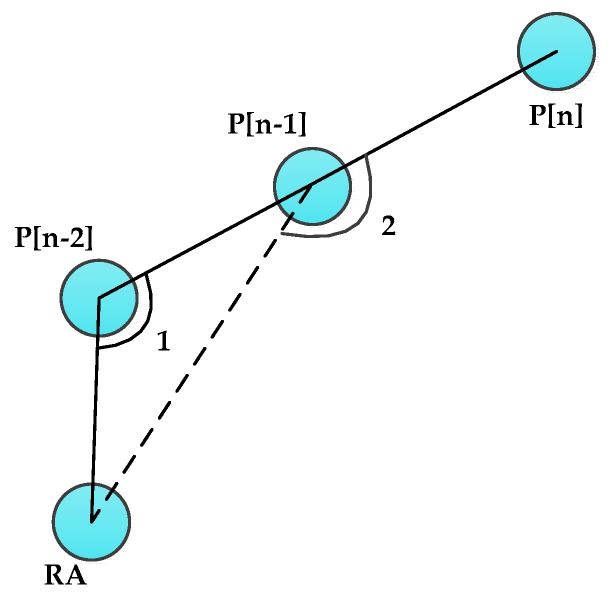
Trajectory with a shorter detour.

**Figure 6:**
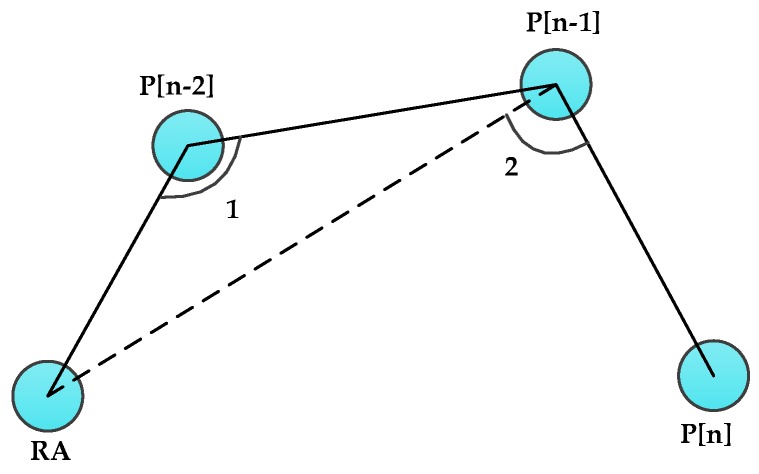
Trajectory with a longer detour.

Subsequently, after the single DAMST angle analysis phase, a further contrastive study among adjacent DAMST angles in the DAMST angle list is executed according to the updated lists. In order to demonstrate the contrastive study, two different cases shown in Figure 5 and Figure 6 are analyzed.

In Figure 5, angles 1 and 2 are two adjacent DAMST angles in the DAMST angle list, representing the (n−1)th and (n−2)th elements in it. It can be seen that if angle 2 is greater than angle 1, the path constructed by P[n], P[n−1] and P[n−2] is smooth, no matter whether angle 2 is a clockwise angle or an anticlockwise angle. From this, if 90° < angle 1 < angle 2, it is assumed that the detour through P[n], P[n−1] and P[n−2] is relatively shorter and P[n−1] is supposed to be reserved in the DAMST point list. Otherwise, as shown in Figure 6, 90° < angle 2 < angle 1, the detour through P[n], P[n−1] and P[n−2] is assumed relatively longer than that in Figure 5, it is hereby that P[n−1] needs to be cancelled from the DAMST point list.

Through comparing the adjacent DAMST angles in the DAMST angle list, more DAMST points on longer detours are cancelled. At the end of each round of the adjacent DAMST angle analysis, the DAMST angle list also needs to be updated accordingly, through recalculating DAMST angles according to the reserved nodes in the DAMST point list. Subsequently, the next round of DAMST angle analysis starts. The DAMST angle analysis is performed from one round to another until there is no more detour can be found.

**Figure 7:**
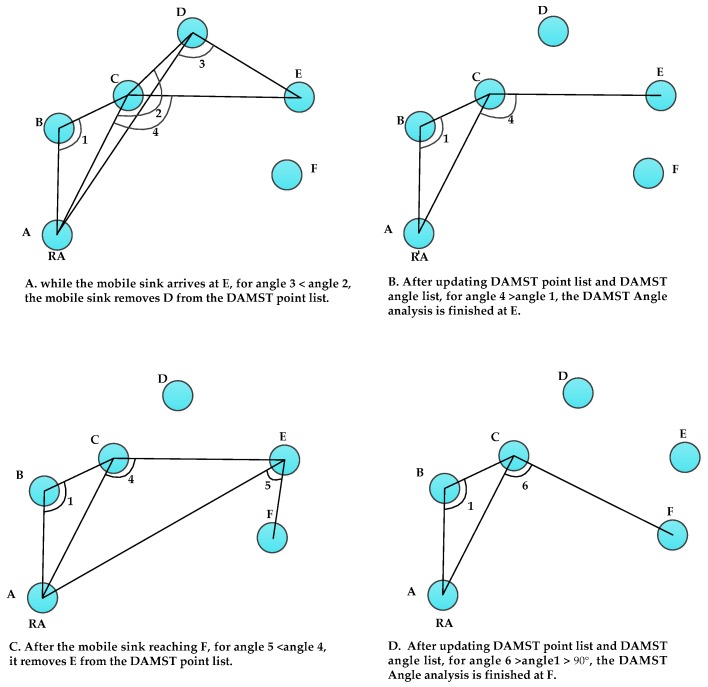
An example of adjacent DAMST angle analysis.

We now use a simple example shown in Figure 7 to illustrate how to cancel DAMST points on detours in the adjacent DAMST angle analysis phase.

In Scenario A, once the mobile sink arrives at E, it appoints E as a footprint and automatically puts E into a DAMST point list. Meanwhile, the mobile sink calculates a new DAMST angle (angle 3) and puts it into DAMST angle list. After this step, the DAMST point list includes nodes B, C, D and E, and the DAMST angle list includes angle 3, angle 2 and angle 1 (all three of these angles are greater than 90°). According to the adjacent DAMST angle analysis, the mobile sink needs to cancel D from the DAMST point list for angle 3 < angle 2.

After canceling D, the mobile sink updates its DAMST point list (including nodes B, C and E) and DAMST angle list (including angle 4 and angle 1, both of them are greater than 90°). For angle 4 > angle 1 > 90°, the DAMST angle analysis is finished at E, as shown in Scenario B. The remaining nodes in the updated DAMST point list are B, C and E, which can be used to establish a detour-aware path A-B-C-E from RA(A) to E, which is shorter than A–B–C–D–E.

In Scenario C, the mobile sink node chooses F as a footprint when approaching F, and updates its DAMST point list to include B, C, E and F, meanwhile updates the DAMST angle list to include angle 1, angle 4 and angle 5. Similarly, for 90° < angle 5 < angle 4, the mobile sink deletes E from the DAMST point list. Subsequently, the mobile sinks updates its DAMST angle list to include angle 1 and angle 6 as well as DAMST point list to include B and C, as shown in Scenario D. For angle 6 > angle 1 > 90°, the DAMST angle analysis is finished at F. Through the DAMST angle analysis, the nodes D and E on detours are cancelled and a better data transmission path A–B–C–F, is established from RA to F.

The whole DAMST angle analysis process is shown in Algorithm 1. After each movement, the mobile sink puts the new footprint and new DAMST angle into the DAMST point list and the DAMST angle list, respectively, and then executes the single DAMST angle analysis and adjacent DAMST angle analysis repeatedly, to cancel the useless footprints on detours, until no more detours are found. The DAMST point list updated by Algorithm 1 should be included in the query generated by the mobile sink for guiding the query forwarding.
**Algorithm 1.** The Damst angle analysis algorithm.1. **Input** P and A;*// Input the DAMST point list and the DAMST angle list*2. $S_{A}$*=* sizeof (A); m = 1;*// Obtain the size of DAMST angle list*3. ***while***(m≠0)// *Start DAMST angles analysis*4.  ｛   j = 0 ;
5.     ***for*** ( i = $S_{A}$; i≥1 ; i - - )// *Start the single DAMST angle analysis phase*6.     ｛
7.       ***if***** ( A[i] < 90° )*//* A[i] < 90° *means a longer detour through P[i]*8.       cancel P[i] ; j + + ;*// Cancel P[i] from the DAMST point list*9.         ***end if***// *Finish the single DAMST angle analysis phase*10.      ｝
11. Update P and A ; $S_{A}$= sizeof (A) ; k = 0 ; 
//*Update the DAMST point list and the DAMST angle list*12.  ***for*** ( i =$S_{A}$; i≥2 ; i - -)// *Start adjacent DAMST angles analysis phase*13.     ｛
14.   
  ***if*** ( A[i] < A[i-1] )//A[i] < A[i-1]
*means a longer detour through P[i]*15.   
  cancel P[i] ; k + + ;// *Cancel P[i] from the DAMST point list*16.   
   
    ***end if***//*Finish adjacent DAMST angle analysis phase*17.       ｝
18.  Update P and A ; $S_{A}$= sizeof ( A ) ; m =$S_{A}$; 
//*Update the DAMST point list and the DAMST angle list*19.   ***if*** ( j = 0 && k = 0 )
20.   m = 0 ;
21.   ***end if***// *Finish the DAMST angle analysis*22. ｝


### 3.4. Routing Algorithm based on Route Deviation Angles

**Figure 8:**
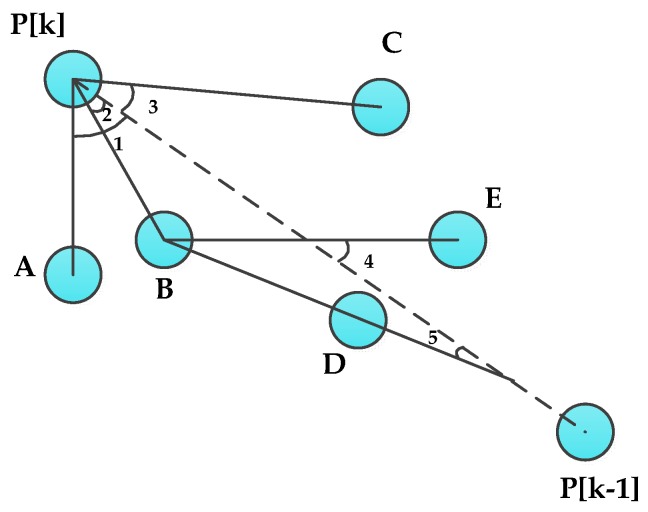
Route deviation angles.

Algorithm 1 cancels the useless footprints on detours from the DAMST point list. In forwarding the query from the mobile sink to the RA, it may happen that the distance between two adjacent DAMST points is over the node communication radius. In order to find a shortest path between these two unconnected adjacent DAMST points, Algorithm 2 is designed. In Algorithm 2, no matter whether the current node is a DAMST point or not, it needs to find a neighbor as a relay node to forward the query, according to its neighbors’ route deviation angles and the next DAMST point the query will go to. The route deviation angle is defined in Definition 2 and depicted in Figure 8.

**Definition 2.**  The route deviation angle of a neighbor node is an angle that is between the edge from the current node to the next DAMST point and the edge from the current node to the neighbor node.

As shown in Figure 8, the current node is the DAMST point P[k], the next DAMST point is the DAMST point P[k−1], the neighbor nodes of P[k] are nodes A, B and C, route deviation angles of the neighbor nodes are respectively angle 1 , angle 2 and angle 3. Through observation of Figure 8, it can be seen that the neighbor having a smaller route deviation angle brings a shorter detour. So the neighbor having the minimum route deviation angle could be chosen as the next relay node. While choosing the next relay node, all neighbors of the current node are put into a neighbor list and all route deviation angles of these neighbors are calculated and then put into a route deviation angle list.

Algorithm 2 is executed according to the neighbor list and the route deviation angle list. In Algorithm 2, if the current node finds that the next DAMST point is in its neighbor list, it would transmit the query to the next DAMST point directly. Otherwise, it chooses a neighbor having the minimum route deviation angle as the next relay node. Furthermore, if this next relay node still can not find out the next DAMST point in its neighbor list, it executes Algorithm 2 according to its own neighbor list and route deviation angle list.

For example, in Figure 8, because P[k−1] is not a neighbor of P[k], and angle 2 < angle 3 < angle 1, P[k] chooses B who has the minimum route deviation angle as a relay node. Then B also can not find P[k−1] in its neighbor list, so it chooses D as its next relay node for angle 5 < angle 4.

To make sure the RA can transmit data back after receiving the query, the mobile sink also generates a route list in the query. The route list is then updated in forwarding the query by Algorithm 2. Specifically, a node is added into the route list once be chosen as a relay node.
**Algorithm 2.** The routing algorithm based on route deviation angles.1. ***Input*** P[n] and R[i]******
***/***/ *P[n] is the next DAMST point, R[i] is the next node in the route list*2. ***if*** ( P[n]∈$N_{c}$)// *If the next DAMST point P[n] is in the neighbor list of the current node*******3.   R[i] = P[n]; n - - ; i + + ;// *Add P[n] into the route list*4.   transmit the query to P[n];// *Transmit the query to P[n]*5. ***else***// *If the next DAMST point P[n] is not in the neighbor list of the current node*6.   calculates ***DA***;// *Calculate route deviation angle list of the current node’ neighbors*7.   R[i] =
$N_{c}^{0}$; i + + ;   // *Add the neighbor node*
$N_{c}^{0}$
*having the minimum route deviation angle into the route list*8. transmit the query to$N_{c}^{0}$;***//***
*Transmit the query to*$N_{c}^{0}$

## 4. Performance Evaluation

### 4.1. Simulations with Different Trajectory Patterns

**Figure 9:**
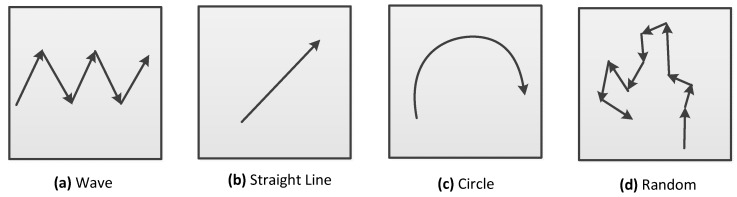
Trajectory patterns.

To examine how the trajectory patterns of mobile sinks affect the performances of DAMST, we designed four kinds of trajectory patterns (Wave, Straight Line, Circle and Random), shown in Figure 9, which are different from each other due to specified changes of the movement deviation direction and the movement deviation angle of the mobile sinks.

In the Wave pattern (a), the mobile sink changes its movement deviation direction from clockwise to anti-clockwise every 20 min with a 60° movement deviation angle. In the Straight Line pattern (b), the mobile sink just moves along a straight line. In the Circle pattern (c), the mobile sink makes a clockwise deviation every 10 min with 60° movement deviation angle. In the Random pattern (d), the mobile sink randomly changes its movement deviation direction every 20 min with a 60° movement deviation angle. In the simulation of each pattern, the mobile sink designates a node near its initial point as a RA.

**Figure 10:**
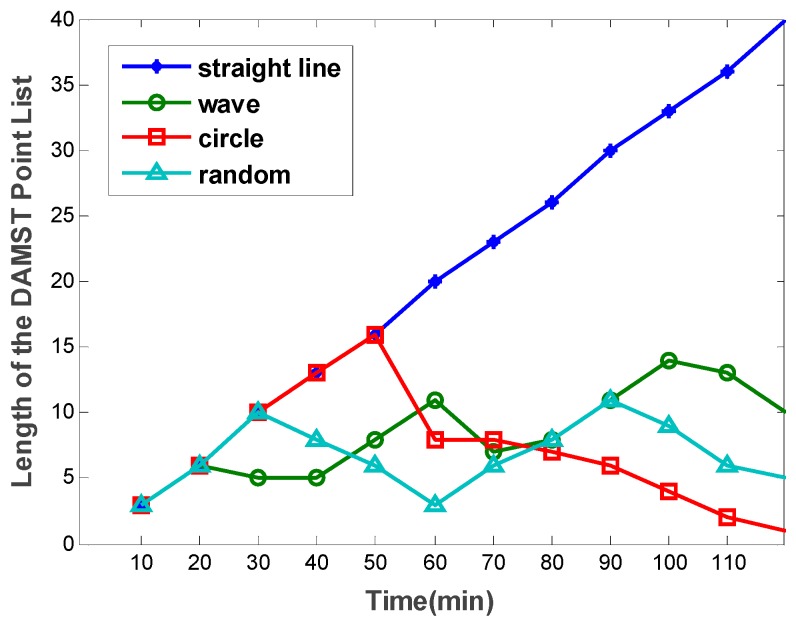
Length of the DAMST point list in the four trajectory patterns.

The length of the DAMST point list denotes the number of nodes in it. Simulation results on the length of the DAMST point list in the four patterns are shown in Figure 10. It can be seen that, only in the Straight Line pattern, the length of the DAMST point list increases continuously as the mobile sink moves, for the reason that all footprints on the trajectory of the mobile sink become DAMST points during the moving time. In the Random pattern, the number of nodes in the DAMST point list changes randomly due to its random trajectory. In the Wave pattern, for the mobile sink changes its movement deviation direction periodically, the length of the DAMST point list also changes accordingly. In the Circle pattern, the length of the DAMST point list increases at first then decreases to zero as the mobile sink moving back to its original place after a period of time.

### 4.2. Effect of Footprint Appointment Interval

The interval to appoint footprints directly affects the number of DAMST points and distance between two adjacent footprints, which further influences the energy consumption and overhead in DAMST. To provide a vivid evaluation about effect of the footprint appoint interval, DAMST with different footprint appointment intervals is performed. In the simulation, each mobile sink moves in the random pattern, the interval from designating a RA to sending a data query is 30 min, and the period of each simulation run is also 30 min. Simulation results for each footprint appointment interval are averaged after taking 20 simulation runs.

**Figure 11:**
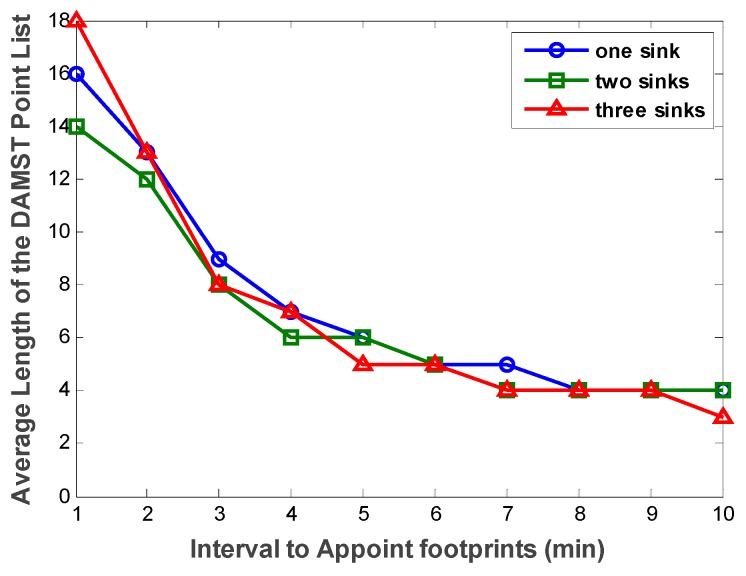
Average length of the DAMST point list with different footprint appointment intervals.

Figure 11 shows the effect of footprint appointment interval on the length of the DAMST point list. The drop-down curves indicate the greater the footprint appointment interval, the smaller the average number of nodes in the DAMST point list. This trend does not change with the variance of number of mobile sinks.

**Figure 12:**
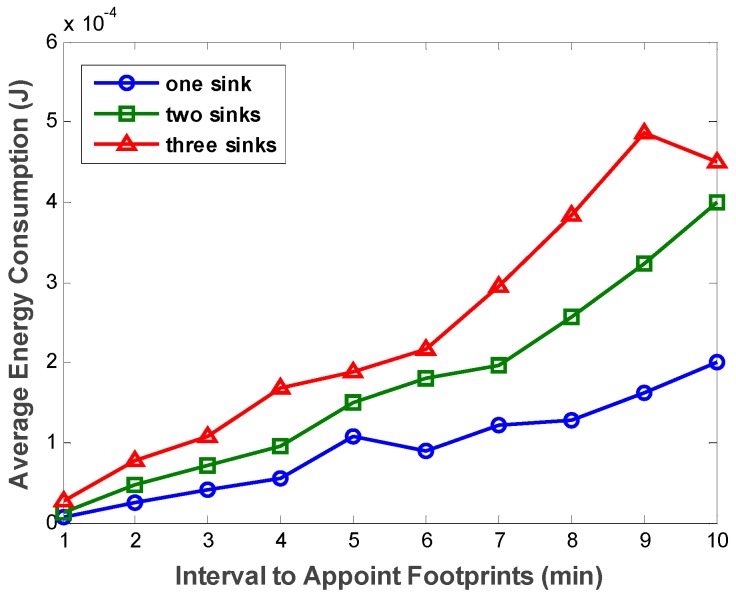
Average energy consumption with different footprint appointment intervals.

However, as the footprint appointment interval increases, the distance between two adjacent DAMST points becomes longer. If the distance between the two adjacent DAMST points is less than node communication radius, the energy consumption on communication is proportional to square of the distance. And if the distance is over the communication radius, Algorithm 2 needs to be executed, in which more energy is consumed on constructing a shortest path. Either way, the increasing of the footprint appointment interval directly leads to the increasing of the distance between two adjacent DAMST points, which indirectly incurs more average energy consumption in network. Figure 12 exactly confirms our analysis. The average energy consumption on communication bulges exponentially with the increasing number of mobile sinks.

**Figure 13:**
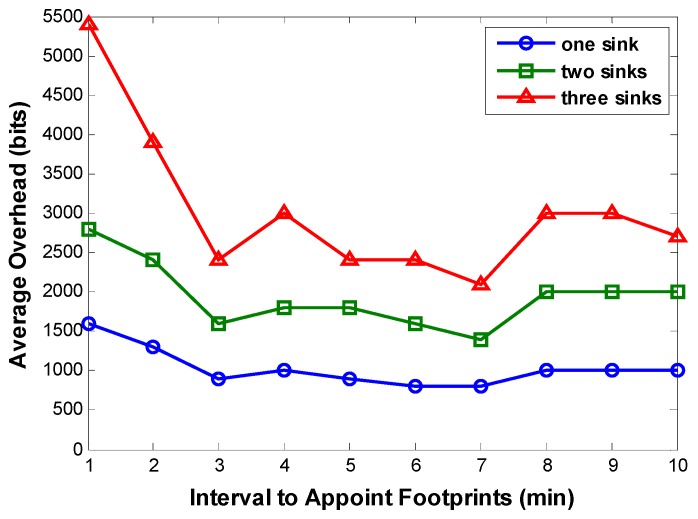
Average overhead with different footprint appointment intervals.

While determining an appropriate footprint appointment interval, its effect on both average energy consumption and average overhead should be considered. Figure 13 shows us how the footprint appointment interval affects the average overhead. As the footprint appointment interval gets longer, the average overhead decreases firstly then increases. On the decrease part, it can be clearly explained: Appropriately extending the footprint appointment interval results in fewer footprints and shorter DAMST point list, which further cause less overhead in Algorithm 1. And in this case, the distance between two adjacent DAMST points becomes longer but still within node communication radius, which is also beneficial for reducing overhead in Algorithm 2.

**Figure 14:**
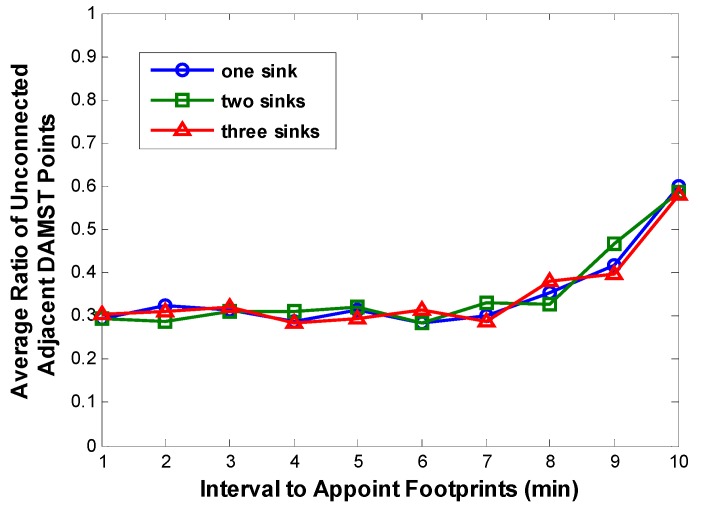
Average ratios of unconnected adjacent DAMST points with different footprint appointment intervals.

We can also make an inference on the increase part: excessively extending footprint appointment interval may produce more unconnected adjacent DAMST points, which further causes more overhead in Algorithm 2. Simulation results on the ratio of unconnected adjacent DAMST points in the DAMST point list are displayed in Figure 14. As expected, the ratio of unconnected adjacent DAMST points becomes greater as the footprint appointment interval is prolonged, which incurs more overhead in finding a shortest path between any two unconnected adjacent DAMST points. Besides, in Figure 13, it is obviously that more mobile sinks brings heavier overhead in network.

### 4.3. Comparison between DAMST and SinkTrail

**Figure 15:**
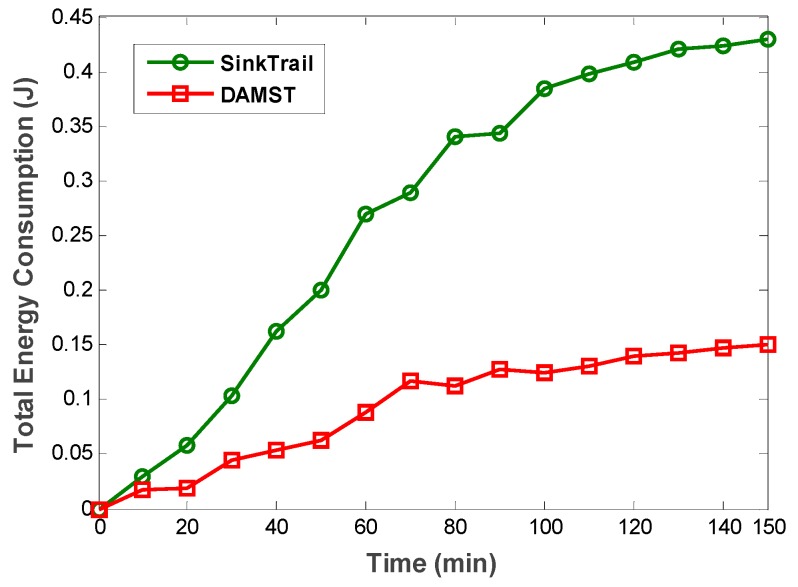
Total energy consumption in DAMST and SinkTrail.

**Figure 16:**
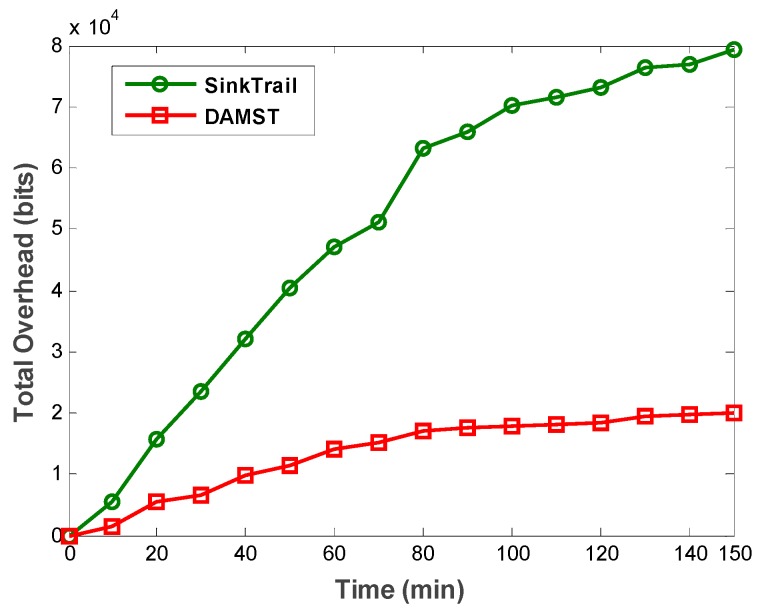
Total overhead in DAMST and SinkTrail.

To prove the effectiveness of DAMST, we compare DAMST with SinkTrail on energy consumption, lifetime and overhead, respectively, in a random pattern. The scenario is set in a 500 m × 500 m region with one mobile sink and 2500 sensor nodes. The interval to appoint footprints is set to 3 min. The movement speed of mobile sink is set to 5 m/min. The communication radius of each node is set to 50 m. It is shown in Figure 15 that DAMST outperforms SinkTrail in energy efficiency, the reason being that, unlike SinkTrail, DAMST does not need to consistently broadcast the location information of mobile sinks. Propagating trail messages and updating tail references in SinkTrail brings considerable overhead in the network. However, DAMST produces less overhead via forwarding packets in the routing path established by the DAMST points and relay nodes. It has been verified in Figure 16 that the overhead of DAMST is much less than that in SinkTrail.

**Figure 17:**
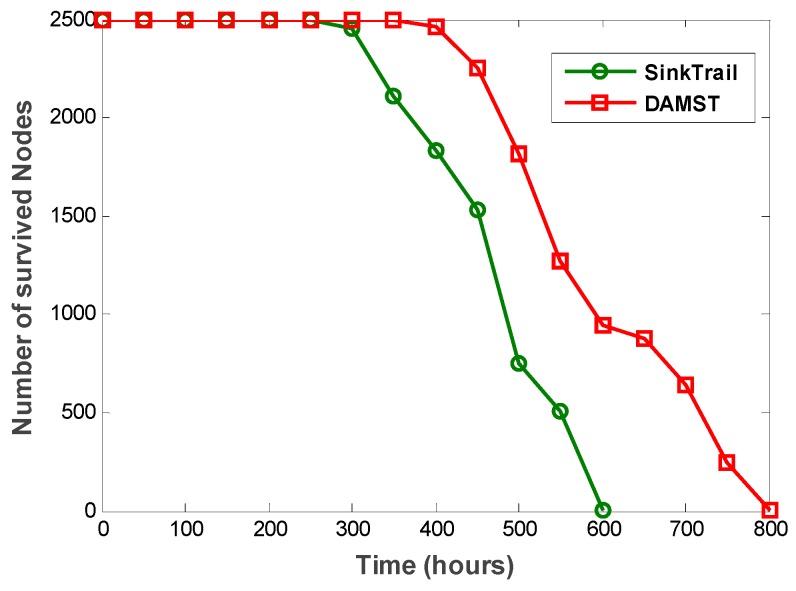
Numbers of survived nodes in DAMST and SinkTrail.

From the above comparison results, it can be seen that DAMST is able to prolong network life time due to its less overhead and energy consumption. Based on this, we further compare DAMST and SinkTrail in a simulation scenario with same parameters at the same period of time and record the number of survived nodes in the entire process. The simulation result in Figure 17 shows that, compared with SinkTrail, the DAMST delays the first node death time and extends the network lifetime.

## 5. Conclusions

In this paper, DAMST is proposed to track mobile sinks via analysis of movement angle changes, in which RAs are appointed to collect data around, record trajectories of the mobile sinks via footprints, and store movement angles in a DAMST angle list. Footprints on detours are eliminated by comparing adjacent movement angles. The remaining footprints are reserved in a DAMST point list to construct energy efficient paths from the RAs to the mobile sinks. Moreover, a routing algorithm is designed to construct a shortest path between two unconnected adjacent DAMST points in the DAMST point list, by utilizing route deviation angles information.

For evaluating the performance of DAMST, a series of simulations are performed, where key factors such as trajectory patterns, footprint appointment intervals, and number of mobile sinks and their effects on DAMST are discussed, which provides guidelines for practical applications. The comparison of DAMST and SinkTrail validates the advantages of DAMST in reducing energy consumption and overhead, as well as prolonging network lifetime.

Our current work includes mobile sink tracking, delay tolerant data collection mechanism with mobile sinks, *etc.* One future direction of this work is how to control movement of multiple mobile sinks to make them collect data cooperatively, and to apply an energy consumption model considering both transmission and computing energy consumptions [19].

## Figures and Tables

**Table 1:** Packet format.

Packet Type		Sink ID	
Sink coordinate		RA ID	
Node Collection radius	Collection period	Data type	Node ID
DAMST point list			
Route list			
Data			

**Table 2:** Definition of notations.

Notations	Definition
*P*	The DAMST point list
*A*	The DAMST angle list
$S_{A}$	Size of the DAMST angle list
$N_{c}$	The neighbor list
$N_{c}^{0}$	The neighbor having the minimum route deviation angle
*DA*	Route deviation angle list of neighbors
*R*	The route list
